# PINK1 Defect Causes Mitochondrial Dysfunction, Proteasomal Deficit and α-Synuclein Aggregation in Cell Culture Models of Parkinson's Disease

**DOI:** 10.1371/journal.pone.0004597

**Published:** 2009-02-26

**Authors:** Wencheng Liu, Cristofol Vives-Bauza, Rebeca Acín-Peréz-, Ai Yamamoto, Yingcai Tan, Yanping Li, Jordi Magrané, Mihaela A. Stavarache, Sebastian Shaffer, Simon Chang, Michael G. Kaplitt, Xin-Yun Huang, M. Flint Beal, Giovanni Manfredi, Chenjian Li

**Affiliations:** 1 Department of Neurology and Neurosciences, Weill Medical College of Cornell University, New York, New York, United States of America; 2 Department of Neurological Surgery, Weill Medical College of Cornell University, New York, New York, United States of America; 3 Department of Physiology, Weill Medical College of Cornell University, New York, New York, United States of America; 4 Judith P. Sulzberger M.D. Columbia Genome Center and Department of Physiology and Cellular Biophysics, Columbia University, New York, New York, United States of America; National Institutes of Health, United States of America

## Abstract

Mutations in PTEN induced kinase 1 (PINK1), a mitochondrial Ser/Thr kinase, cause an autosomal recessive form of Parkinson's disease (PD), PARK6. Here, we report that PINK1 exists as a dimer in mitochondrial protein complexes that co-migrate with respiratory chain complexes in sucrose gradients. PARK6 related mutations do not affect this dimerization and its associated complexes. Using *in vitro* cell culture systems, we found that mutant PINK1 or PINK1 knock-down caused deficits in mitochondrial respiration and ATP synthesis. Furthermore, proteasome function is impaired with a loss of PINK1. Importantly, these deficits are accompanied by increased α-synclein aggregation. Our results indicate that it will be important to delineate the relationship between mitochondrial functional deficits, proteasome dysfunction and α-synclein aggregation.

## Introduction

Parkinson's Disease (PD) is a neurodegenerative disorder with pathological hallmarks of dopaminergic neuron degeneration in the substantia nigra pars compacta, and cytoplasmic inclusion Lewy bodies that contain mainly α-synuclein aggregates. The PD pathogenic pathways involve defects in many cellular processes such as protein degradation, oxidative stress, phosphorylation signaling, and mitochondrial function.

Mitochondria are complex subcellular organelles that play diverse and critical roles in energy production, pyrimidine biosynthesis, fatty acid metabolism, calcium homeostasis, oxidative stress response and apoptotic cell death. Mitochondrial dysfunction is implicated in normal as well as pathological aging, especially in many neurodegenerative diseases such as Alzheimer's disease, Huntington's disease, Amiotrophic Lateral Sclerosis and PD [1], [2], [3]. In idiopathic PD patients, a 30–40% decrease of mitochondrial electron transport chain (ETC) complex I activity was observed in platelets, skeletal muscle and brain [4], [5], [6], [7]. Moreover, complex I inhibitors 1-methyl-4-phenyl-1,2,3,6-tetrahydropyridine (MPTP) and rotenone induce a clinical syndrome that replicates the hallmarks of PD in human, non-human primates and rodents [8], [9], [10], [11]. Remarkably, mitochondria are also implicated in most genetic forms of familial PD: ETC Complex IV activity is reduced in an α-synuclein mouse model [12]; the mitochondrial respiratory capacity is decreased and oxidative damage is increased in both Parkin-knockout mouse and *Drosophila* [13], [14]; a portion of DJ-1 and LRRK2, two PD-associated proteins, localize to mitochondria [15], [16]; overexpression of DJ-1 is protective against oxidative stress and mitochondrial damage, while loss of DJ-1 is harmful [16], [17]. Despite this wealth of evidence, it is still unclear whether mitochondrial dysfunction is a primary causal event or a secondary consequence of PD.

The most direct evidence indicating a primary role of mitochondria in PD pathogenesis comes from the identification of PINK1 as the causal gene for PARK6, an autosomal recessive form of PD. PINK1 is a Ser/Thr kinase localized in mitochondria [18], [19], [20]. Thus, the primary causal event in PARK6 has to be associated with mitochondria. Recent studies have begun to shed light on the mechanisms through which PINK1 causes PD, and implicate cellular functions such as mitochondrial respiration [21], [22], mitochondrial morphology and dynamics [23], [24], [25], [26], [27], and oxidative stress [21], [28]. Our study directly addresses the consequences of PINK1 defects on mitochondrial function, and on cellular abnormalities that are important to PD. We report here that in cell culture systems, mutant PINK1 or loss of PINK1 induces mitochondrial dysfunction, defective proteasome function and increases α-synuclein aggregation.

## Materials and Methods

### Plasmid and viral constructs

Full length PINK1 cDNA was cloned into a pshuttle-1-3XFlag-IRES-GFP vector (Stratagene) to generate Flag-tagged PINK1. PINK1 cDNA carrying Del 245, L347P or E417G mutation was made by staggered PCR. To make adenoviruses, wild type-, Del 245-, L347P- or E417G-PINK1-3XFlag-IRES-EGFP in pshuttle-1 vector were used to re-combined into pAdeasy (Stratagene). These viral constructs were transfected into HEK293 cells to obtain PINK1 recombinant viruses, followed by virus amplification and purification based on the manufacture's protocol. Viral titers were determined by method previously described [29]. To generate various V5 tagged PINK1, wild type and mutant PINK1 cDNAs were cloned into a pcDNA3.1 vector (Invitrogen).

### Transfection and immuno-precipitation

DNA was transfected into cells using Lipofectamine 2000 according to the manufacturer's protocol. 48 hours post transfection, cells were harvested and re-suspended in lysis buffer (50 mM Tris, pH 7.4, 150 mM NaCl,1 mM EDTA and 0.1% Triton-100) supplemented with 2× protease inhibitor (Roche) and homogenized with dounce homogenizer. The cell homogenates were then centrifuged at 11,000 g for 10 min, and cell lysates were collected. For immuno-precipitation, cell lysates were first pre-cleared with protein A & G agarose beads for one hour at 4°C, and then incubated with rabbit anti-Flag or rabbit anti-V5 Ab for at least 2 hours at 4°C with constant agitation, followed by four washes of 20 min each with the lysis buffer. The beads that captured PINK1 complexes were mixed with equal amount of 2× SDS sample buffer and heated at 95°C for 10 min to elute the complex proteins. Eluents were used for SDS-PAGE, followed by Western analyses with mouse anti-V5 (Invitrogen) or mouse anti-Flag antibody (Ab, Sigma). Human PINK1 antibodies from Novus (BC100-494) were used for the detection of endogenous PINK1.

### Cell lines stably expressing shRNAs against PINK1

DNA oligos encoding for small hairpin RNAs (shRNA) targeting rat PINK1 were annealed and cloned into a rAAV vector. The following oligos were used: PINK1 5′-ATCCCCGCACACTCTTCCTCGTTAT **CTTCCTGTCA** ATAACGAGGAAGAGTGTGCTTTTTGGAA-3′. The gene specific sequences are underlined and the loop from the murine miR-23 is shown in bold. These rAAV expression plasmids were subcloned into a vector containing the neomycin resistance gene. The plasmids were transfected into PC12 cells. Neomycin resistant clones were isolated, expanded and analyzed for PINK1 expression following G418 selection (500 µg/ml).

### Quantitative PCR (Q-PCR)

Real-time PCR was performed using SYBR Green Master Mix (Applied Biosystems) on an ABI Prism 7000 Sequence Detection System (Applied Biosystems). The level of PINK1 transcript was normalized against GAPDH.

### Infection of SH-SY5Y cells with recombinant adenovirus

Purified recombinant adenoviruses (50 particles per cell) were used to infect SH-SY5Y cells cultured in DMEM/F12 (1∶1) medium containing 10% fetal bovine serum. 48 hours post infection, cells were harvested for further experiment.

### Measurements of O_2_ consumption

Oxygen consumption in intact cells was measured in a 300-µl reaction chamber equipped with a Clark-type polarographic electrode (Hansatech Instrument, UK) as described previously (Hofhaus, et al., 1996). Briefly, cells were trypsinized, counted in a Z1 automated cell counter (Beckman-Coulter, Miami, FL), and re-suspended at 1.5×10^6^ cells in DMEM containing no glucose and no fetal bovine serum and supplemented with 1 mM sodium pyruvate. After a baseline trace of coupled respiration was recorded with adequate time for accurate slope measurement, the endogenous respiration was completely inhibited by 1.5 mM KCN.

### ATP synthesis measurement

ATP synthesis was measured in digitonin-permeabilized SH-SY5Y with a luciferase-based kinetic assay as previously described [30]. Briefly, cells were collected by trypsinization, pelleted by centrifugation, counted, and resuspended at 1×10^7^ cells/ml in buffer A containing 150 mM KCl, 25 mM Tris-HCl, 2 mM EDTA, 0.1% bovine serum albumin, 10 mM potassium phosphate, 0.1 mM MgCl_2_, pH 7.4. 160 µl of the cell suspension was incubated with 50 µg/ml digitonin for 1 min at room temperature, diluted with 1 ml of buffer A, and centrifuged at 6000 r.p.m. for 1 min. The cell pellet was re-suspended in 160 µl of buffer A, and 0.15 mM diadenosine-pentaphosphate Ap_5_A, 0.1 mM ADP, 1 mM malate, 1 mM pyruvate, and 10 µl of buffer B containing 0.8 mM luciferin and 20 µg/ml luciferase were added. The light emission was recorded by an Optocomp I luminometer (MGM Electronics) at 15-sec intervals for a total recording time of 4 min. A standard ATP/luminescence curve was constructed by measuring the luminescence of different ATP concentrations.

### ATP content and proteasome activity

10 cm plates of HeLa cells were transiently transfected by CFP-degron (CFPde) with Lipofectamine 2000. Cells were plated into a 96-well plate. After a 1 hr pre-incubation of cells in glucose-free DMEM, cells were placed in glucose-containing media, but treated with 0 to 6 mM of 2-deoxyglucose (2-DG) for 5 hrs. Proteasome activity was indirectly assessed in half of the wells of cells by the degradation of CFPde as previously described [31]. The other half of the cell wells of the 96-well plate were used to measure ATP content using the luminescent ATP Assay Kit (Calbiochem), for which the release of luminescence reflects the breakdown of ATP.

### Assays for protesome activity affected by PINK1

*CFP-degron method*: 24-well plate of HeLa cells were transfected with a CFP-degron construct (CFPde) alone or co-transfected with 10 nM siSCRAMBLE sequence (siSCR) or 10 nM siRNA directed against PINK1 (Dharmacon), with Lipofectamine 2000. 24 hrs post transfection, cells were plated into 96 well format. 48 hrs post transfection, cells that were transfected with CFPde alone were split and half of it were treated with 1 uM MG-132, a proteasome inhibitor, for 24 hrs. All cells were fixed, stained with Hoechst 333342 for nuclei, and scanned on the automated confocal INCELL Analyzer (INCA) 3000, as previously described [31]. The fluorescent intensity of the cytoplasm of individual cells was assessed using the object intensity module. SiRNA sequences: GAGAGGUCCAAGCAACUA TT and CCUGGUCGACUACCCUGAU TT.

*Suc-LLVY-AMC method*: 5×10^5^ SH-SY5Y cells were infected with adenovirus expressing wild type, L347P and E417G-PINK1. Three days post infection, cells were collected, re-suspended with a buffer containing 10 mM HEPES, pH 7.4 and 0.5 mM MgCl_2_, and homogenized with dounce homogenizer. Chymotrypsin activity was measured as described by Ehlers [32]. In control experiments, the proteasome was inhibited by addition of 50 µM MG 132 to cell lysates 10 min before adding the fluorogenic substrate. The assay was performed in a 96 cell plate; and fluorescence was measured 1.5 hours after incubation on an HTS 7000 plus fluorescent plate reader (PerkinElmer, Boston, MA) with excitation and emission wavelengths of 360 and 465 nm, respectively. The same method was also applied to PC12 cells with reduced PINK1 expression.

### Filter trap assay

SH-SY5Y cells or stable HeLa cell lines expressing wild type or A53T α-synuclein were transfected with siRNA against PINK1. Two days post transfection, cell pellets were collected and lysed with a buffer containing 20 mM Tris, pH 7.5, 1% Triton X-100, 1 mM EDTA, 1 mM DTT and 20 U/ml RQ1 RNase Free DNase (Promega). 100 µg proteins from lysates were mixed with SDS to a final concentration of 1% SDS. SDS insoluble and soluble fractions in the lysates were separated with a modified filter trap assay by co-filtration through two filters, a cellulose acetate membrane on top to capture SDS insoluble proteins and a PVDF membrane underneath to capture SDS soluble proteins. The cellulose acetate membrane was probed with anti-GFP Ab, whereas the PVDF membrane was incubated with anti-actin Ab as a control for protein extraction and loading.

### Statistical analysis

Differences between mutant PINK-1 cell lines and KO PINK-1 cells and their respective controls were assessed by ANOVA and by the nonparametric Kruskal-Wallis test. Paired genotype differences were assessed by the post-hoc Fisher's PLSD test. All test and calculations were done with the statistical package StatView 5.0 for PC (SAS Institute).

## Results

### The PINK1 constructs and expression

**Figure 1:**
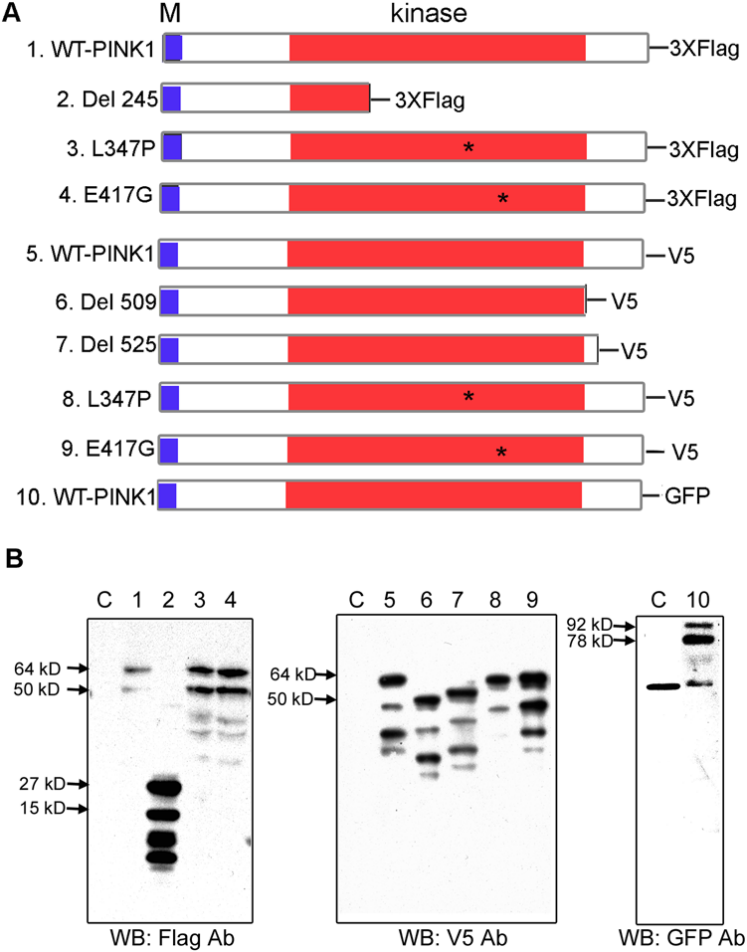
PINK1 constructs and their expressions. A) A schematic depiction of PINK1 constructs. Full length wild type, L347P-, or E417G- PINK1 tagged with Flag, V5, or GFP are indicated. Several truncated PINK1 tagged with Flag or V5 are also depicted. M stands for mitochondrial targeting sequence. B) Confirmation of the expression of the above constructs in HEK 293 cells. HEK293 cells were transfected by various PINK1 constructs, and their lysates were analyzed by Western blots with Flag antibody (Left panel), V5 antibody (the middle panel) or GFP antibody (the right Panel). Lane 1–10 are lysates from cells transfected by plasmids with the same numbering as shown in A). The lysates from the cells transfected with the empty cloning vector without PINK1 insert were used as controls (labeled as C). The results demonstrated that the expression of all constructs yielded recombinant PINK1 proteins with expected molecular weights.

PINK1 has a mitochondrial targeting signal, followed by a conserved Ser/Thr kinase domain, and a C-terminal domain. PD-causing mutations have been found in both the kinase and the C-terminal domains [18], [19], [20]. Among these mutations, G309D, L347P and E417G are on amino acid residues that are well conserved throughout evolution and thus are expected to be important for PINK1 kinase function. Another human disease-causing mutation is a large deletion of PINK1 after amino acid 245 (Del 245), which eliminates most of the kinase domain. Therefore, we introduced the mutations L347P, E417G, and Del 245 into human PINK1 cDNAs, and then cloned into mammalian expression plasmid vectors. In order to identify the recombinant proteins, V5, Flag and GFP tags were added to the C-termini (**Fig 1A**). These constructs allowed efficient expression of PINK1 in mammalian cells (**Fig 1B**). It is worth noting that L347P-PINK1, although prone to degradation by some purification methods, was stable in our experimental systems and conditions (**Figure 1B, lane 3 and 8**), in contrast to a previously reported instability of L347-PINK1 [33].

### PINK1 proteins form dimers via the kinase domain

**Figure 2:**
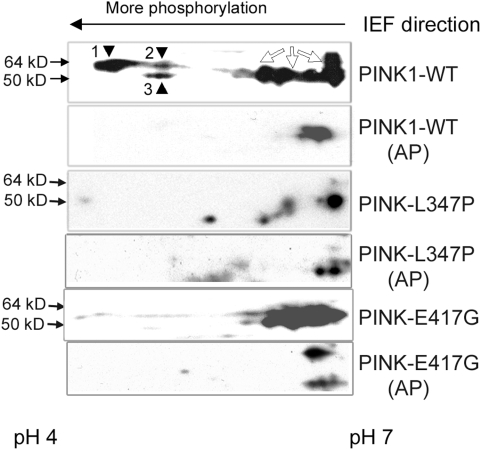
PINK1 auto-phosphorylation is impaired by L347P and E417G mutations. PINK1 protein is auto-phosphorylated in the cells expressing wild type PINK1 and the auto-phosphorylation was decreased in cells expressing L347P- or E417G-PINK1. Lysates from the SH-SY5Y cells expressing wild type-PINK1-Flag (1^st^ and 2^nd^ panels), L347P-PINK1-Flag (3^rd^ and 4^th^ panels) or E417G- PINK1-Flag (5^th^ and 6^th^ panels) were subjected to 2-dimensional gel electrophoresis, followed by Western analyses using anti-Flag Ab. Of the same protein, the more phosphorylated ones migrated to the more acidic end (left hand) of the isoelectric focusing (IEF) gel, while less or non-phosphorylated ones migrated to the more basic end (right hand) of IEF. 1^st^ panel: Wild type PINK1 along the pH gradient of the IEF. Spot 1, 2 (64 kD), and 3 (50 kD) migrated to the acidic end, and several others (open arrows) to the basic end. 2^nd^ panel: A control of wild type PINK1 proteins treated with alkaline phosphotase (AP). While the spots on the basic end still remained in the same region of the gel as in the 1^st^ panel, spot 1, 2, and 3 were missing. This indicated that these 3 spots were phospho-PINK1 in 1^st^ panel, and were de-phosphorylated in the 2^nd^ panel. The difference between spot 1 and 2 reflected the degree of phosphorylation on PINK1. In cells expressing L347P or E417G-PINK1, spot 1, 2 and 3 were greatly reduced, indicating reduced auto-phosphorylation on L347P- or E417G-PINK1 (3^rd^ and 5^th^ panels). The 4^th^ and 6^th^ panels are controls (treated with AP) for the 3^rd^ and 5^th^ panels respectively, similar to the 2^nd^ panel.

**Figure 3:**
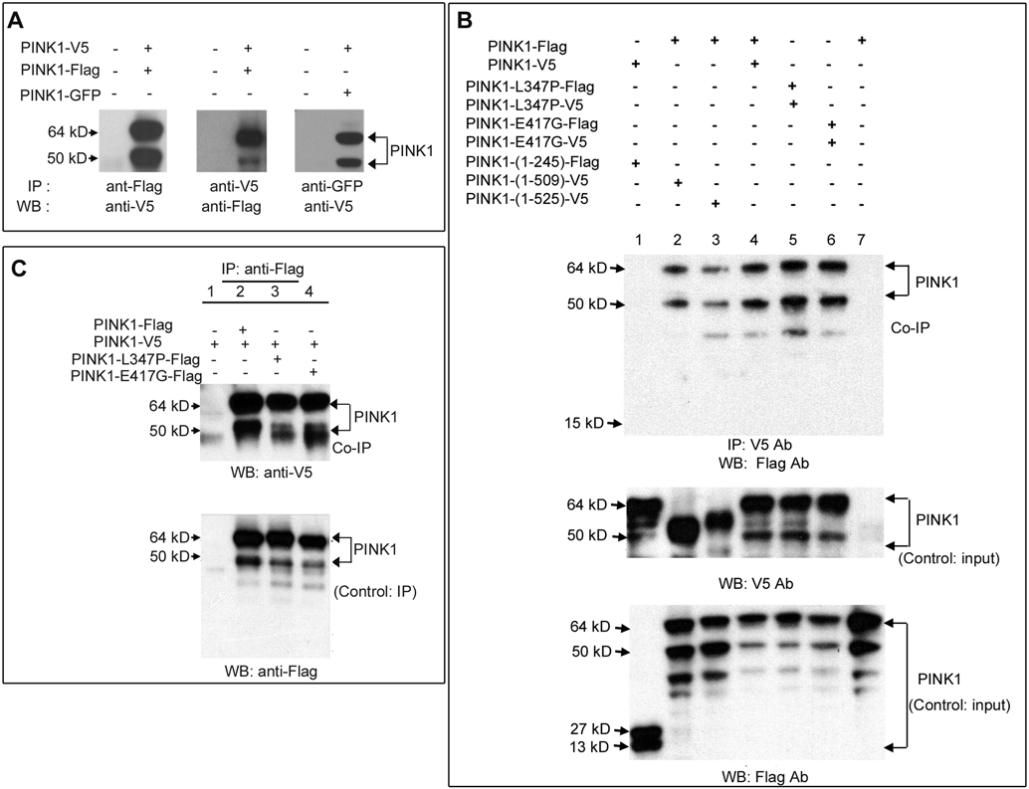
Dimerization of PINK1 via the kinase domain. In all experiments, PINK1 with different tags were co-transfected in pairs into HEK293 cells. A tag was used for immunoprecipitation (IP), and Ab against another tag was used for Western blots (WB). Results were confirmed in COS and HeLa cells (data not shown). A) Wild type PINK1 form dimers. Left lanes in all three panels: lysate from cells without transfection as negative controls; right lanes in all panels: lysate from cells co-transfected with a pair of PINK1 constructs. In the left and middle panels, PINK1-Flag and PINK1-V5 were co-transfected. Anti-Flag Ab could co-IP PINK1-V5 (left panel) and vice versa (middle panel), indicating that PINK1-Flag and PINK1-V5 form a dimer. PINK1 dimerization is confirmed when PINK1-GFP and PINK1-V5 were co-transfected, anti-GFP Ab could co-IP PINK1-V5 (right panel). B) Wild type and mutant PINK1 form homo-dimers via the kinase domain. Lysates from cells expressing PINK1-V5 and PINK1_1-245_-Flag (lane 1), PINK1-Flag and PINK1_1-509_-V5 (lane 2), or PINK1-Flag and PINK1_1-525_-V5 were isolated (lane 3), and subject to Western analysis with V5 antibody (middle panel, input control) or Flag antibody (bottom panel, input control) to confirm the expression of expected tagged recombinant protein. These lysates were then immunoprecipitated with mouse anti-V5 Ab, and subjected to Western analyses with rabbit anti-Flag Ab (upper panel; Co-IP). PINK1_1-245_-Flag abolished dimerization (lane 1), whereas PINK1_1-525_-V5 and PINK1_1-509_-V5 could dimerize normally (lanes 2 and 3). Thus amino acid residues 246–509 are necessary for dimerization. L347P and E417G mutations did not disrupt the PINK1-PINK1 interaction (lane 5 and 6). C) Mutant PINK1 can also form hetero-dimers with wild type PINK. Lysates from cells expressing 1) PINK1-V5, 2) PINK1-V5 and PINK1-Flag, 3) PINK1-V5 and PINK1-L347P-Flag, 4) PINK1-V5 and PINK1-E417G-Flag were isolated and immunoprecipitated with rabbit anti-Flag Ab. The IP and Co-IP fractions were then subjected to Western analyses with mouse anti-V5 Ab (upper panel, Co-IP) or mouse anti-Flag Ab (lower panel, control IP). Wild type PINK1 form dimers (lane 2), and the disease-causing PINK1 mutations did not affect the dimerization (lanes 3, 4).

We confirmed that PINK1 is indeed a kinase, that it is auto-phosphorylated, and that familial mutations impair the kinase activity (**Figure 2**), as others have shown [33]. One of the mechanisms to regulate kinase activity is by homo-dimerization of the kinase [34], [35]. We therefore investigated whether PINK1 also dimerizes, and if disease-causing mutations disrupt this dimerization and impair kinase activity. V5, Flag, and GFP tag were each fused to a wild type PINK1, L347P-PINK1, E417G-PINK1 and Del-245-PINK1. All combinations of the above constructs were transfected in HEK293 cells for co-immunoprecipitation (co-IP) experiments. Co-transfection of PINK1-V5 with PINK1-Flag followed by pull-down of the protein with the anti-Flag antibody and detection by WB with the anti-V5 antibody showed that wild type PINK1 protein exist as a dimer (**Figure 3A, left panel**). The same results were obtained in a reverse direction when the protein was pulled-down with anti-V5 antibody and detected with anti-Flag (**Figure 3A, middle panel**). Co-transfection of PINK1-GFP with PINK1-V5, followed by IP with anti-GFP antibody and detection of PINK1 with anti-V5 antibody also proved the dimerization of PINK1 (**Figure 3A, right panel**). These results were confirmed in COS and HeLa cells (data not shown). To map the domain required for dimerization, we used Del 245, Del 509 and Del 525-PINK1 (**Fig 1A**) for co-IP with the wild type PINK1. Whereas Del 509 and Del 525 dimerized with wild type PINK1 (Fig 3B, lane 2 and 3 of the top panel), Del 245 lost such ability (Fig 3B, lane1 of the top panel). Therefore, the interaction for PINK1 dimerization requires amino acid 246–509 within the kinase domain. A further co-IP experiment with L347P-PINK1 and E417G-PINK1 demonstrated that these mutations do not affect the homo-dimerization of PINK1 kinase (**Figure 3B, lane 5 and 6, top panel**). This implies that the kinase deficit in L347P-PINK1 and E417G-PINK1 is not due to a failure of dimerization. We also showed that L347P-PINK1 and E417G-PINK1 can form hetero-dimers with the wild type PINK1 (**Fig 3C**).

### Recombinant and endogenous PINK1 are associated with protein complexes in mitochondria

**Figure 4:**
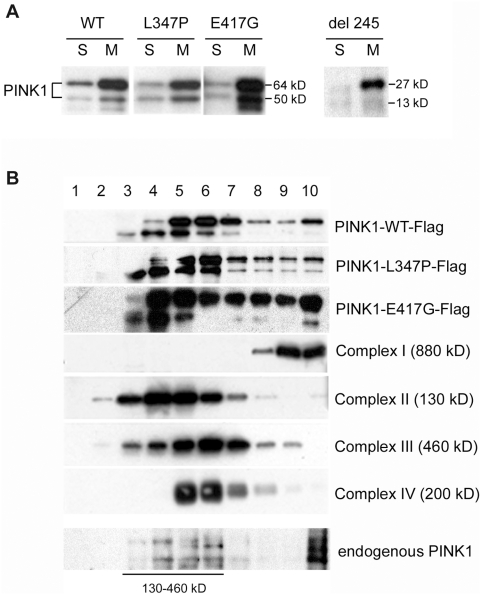
Recombinant and endogenous PINK1 are associated with protein complexes in mitochondria. In all experiments, adenoviruses of PINK1-Flag, L347P-PINK1-Flag, E417G-PINK1-Flag or Del 245 PINK1-Flag were infected into SH-SY5Y cells. A) Western blot analysis of PINK1 sub-cellular distributions with anti-Flag Ab. The two forms of PINK1 (a 64 kD full length protein and a truncated form of 50 kD, presumably a proteolytic product) are present in mitochondria and cytosol. The L347P, E417G or Del 245 mutant PINK1 did not affect this distribution. S: cytosolic fraction; M: mitochondrial fraction. B) PINK1 is associated with protein complexes. Mitochondrial proteins were sub-fractionated by 15% to 35% discontinuous sucrose gradient, from which fractions 1–10 were collected from top (lighter proteins) to bottom (heavier proteins or complexes). They were subjected to SDS-PAGE, and Western analyses with anti-Flag Ab for PINK1 (the top three panels); anti-39 kD protein Ab for complex I (4^th^ panel); anti-70 kD protein Ab for complex II (5^th^ panel); anti-core 2 Ab for complex III (6^th^ panel); and anti-cox I Ab for complex IV (7^th^ panel). No PINK1 was observed in lane 1 and 2, the fractions that contained proteins of the sizes for monomeric PINK1. Instead, PINK1 was associated with protein complexes ranging from 130–900 kD, which co-migrated with ETC complexes. The L347P and E417G mutations did not affect the PINK1 association and distribution of these complexes. More importantly, anti-human PINK1antibodies (Novus) detected endogenous PINK1 in SH-SY5Y cells with similar distribution along the sucrose gradient (bottom panel).

PINK1 has a mitochondrial targeting signal at its N terminal, and was localized to mitochondria in our experimental systems (**Figure 4A**) as in other systems [19], [33]. Since many proteins in mitochondria are compartmentalized and tightly organized in functional multi-protein complexes such as the ETC complexes, we investigated if this is also true for PINK1, and if mutations in PINK1 could impair this association. Using sucrose gradients to sub-fractionate mitochondrial extracts, we demonstrated that indeed wild type PINK1 exists in protein complexes that fractionate with respiratory chain complexes I, II, III, IV, (**Figure 4B**, top panel) and mutations in PINK1 do not alter the localization of the protein within the multi-protein complexes (**Figure 4B**, 2^nd^ and 3^rd^ panels). PINK1 was not detected in lanes 1 and 2 that contain proteins of molecular mass close to that of PINK1 monomers, further suggesting that PINK1 protein does not exist as a monomer in these cells. Furthermore, the endogenous PINK1 migrates within the same fractions as the recombinant proteins along the sucrose gradient (**Figure 4B, bottom panel**), indicating that endogenous PINK1 is present as a dimer and associates with larger protein complexes.

### Mutant PINK1 impairs mitochondrial respiration and ATP synthesis in cultured SH-SY5Y cells

**Figure 5:**
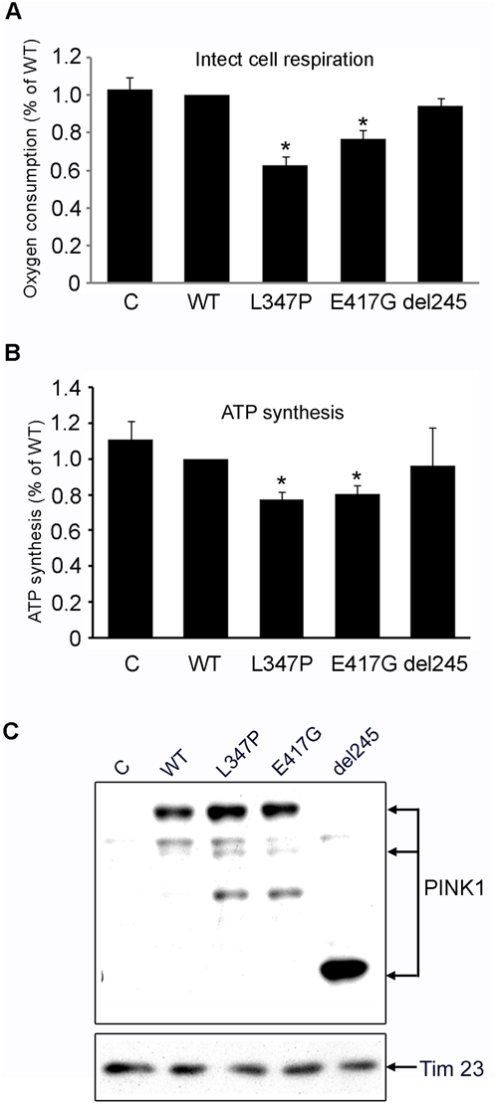
Mutant PINK1 impairs mitochondrial respiration and ATP synthesis in cultured SH-SY5Y cells. A) Oxygraphic measurement of SH-SY5Y cells infected with adenovirus of wild type (WT) PINK1, L347P-PINK1, and Del 245 PINK1. Normal respiration was measured with pyruvate as a substrate in non-infected SH-SY5Y control (C), cells infected with WT, L347P, E417G or Del 245 PINK1. A statistically significant deficit in oxygen consumption was detected in cells expressing L347P-PINK1 compared to wild type PINK1 (37.7% reduction; n = 3, p<0.01, ANOVA) or to non-infected control cells (39.3% reduction; n = 3, p<0.01, ANOVA). In cells expressing E417G-PINK1, oxygen consumption was significantly reduced compared to cells expressing wild type PINK1 (23.1% reduction, n = 3, p<0.05, ANOVA) or to non-infected cells (25.2% reduction, n = 3, p<0.05, ANOVA). There is no significant difference in respiration changes between cells expressing WT-PINK1 and non-infected control cells or between cells expressing WT-PINK1 and Del 245-PINK1. B) Measurements of ATP synthesis with malate/pyruvate as substrates in cultured SH-SY5Y cells. A statistically significant deficit in ATP synthesis was detected in cells expressing L347P-PINK1 compared to wild type PINK1 (23% reduction; n = 15, p = 0.001, Student t test) or to non-infected control cells (30% reduction; n = 5, p = 0.012, student t test). In cells expressing E417G-PINK1, ATP synthesis was significantly reduced compared to cells expressing wild type PINK1 (19.7% reduction, n = 12, p = 0.001, student t test) or to non-infected cells (27.4% reduction, n = 12, p = 0.011, student t test). There is no significant difference in ATP synthesis between cells expressing WT-PINK1 and non-infected control cells (n = 5, p = 0.646, student t test) or between cells expressing WT-PINK1 and Del 245-PINK1 (n = 5, p = 0.849, student t test). C) As controls, lysates used for respiration and ATP synthesis were subsequently subjected to Western analysis with Flag Ab (top panel) and Tim 23 Ab (bottom panel). Results indicate that equal expression of various recombinant PINK1 and equal amount of mitochondria were used for all the experiments.

A cardinal mitochondrial function is to produce energy through oxidative phosphorylation (OXPHOS). Recent studies suggest that the OXPHOS system is regulated by phosphorylation [36], [37], [38], [39], [40]. To determine whether mutant PINK1 affects mitochondrial function, we generated Flag-tagged wild type, L347P, E417G or Del 245 PINK1-adenovirus to infect human neuroblastoma SH-SY5Y cells with high efficiency. We measured cellular respiration 48 hours post-infection. A statistically significant defect in O_2_ consumption rate was observed in intact SH-SY5Y cells expressing L347P and E417G PINK1 using pyruvate as a respiratory substrate, compared to the wild type PINK1 controls (**Figure 5A**). Since SH-SY5Y cells express endogenous PINK1, our data suggest that over-expressed L347P- and E417G-PINK1 exert a dominant negative effect, probably through the formation of hetero-dimers of mutants L347P and E417G with wild type PINK1 as shown in **Fig 3C**. In fact, Del 245-PINK, which lacks the kinase and C-ternimal domains, and consequently cannot form heterodimers with wild type PINK1 (**Fig 3C**), acts as a null control for the dominant negative effect. And as expected, Del 245 PINK1 did not cause a respiratory defect (**Figure 5A**).

One of the major deleterious consequences of a defective ETC is an ATP synthesis deficit that has a wide spread impact on most cellular functions. Therefore, we measured mitochondrial ATP synthesis using the Complex I substrates malate and pyruvate, and demonstrated that cells expressing L347P- and E417G-PINK1 have statistically significant reductions of mitochondrial ATP synthesis, compared to non-infected cells or cells infected with wild type PINK1 (**Figure 5B**). Consistent with the respiration measurements, Del 245-PINK1, as a null control, did not show a defect in ATP synthesis. By controlling and normalizing for various recombinant PINK1 (**Figure 5C, top panel**), and a mitochondrial protein, Tim23 (**Figure 5C, bottom panel**), we conclude that the respiration and ATP synthesis deficit is a functional defect of mitochondria, rather than a depletion of mitochondrial mass, or a variation of transgene expression.

### Loss of PINK1 impairs mitochondrial respiration and ATP synthesis

**Figure 6:**
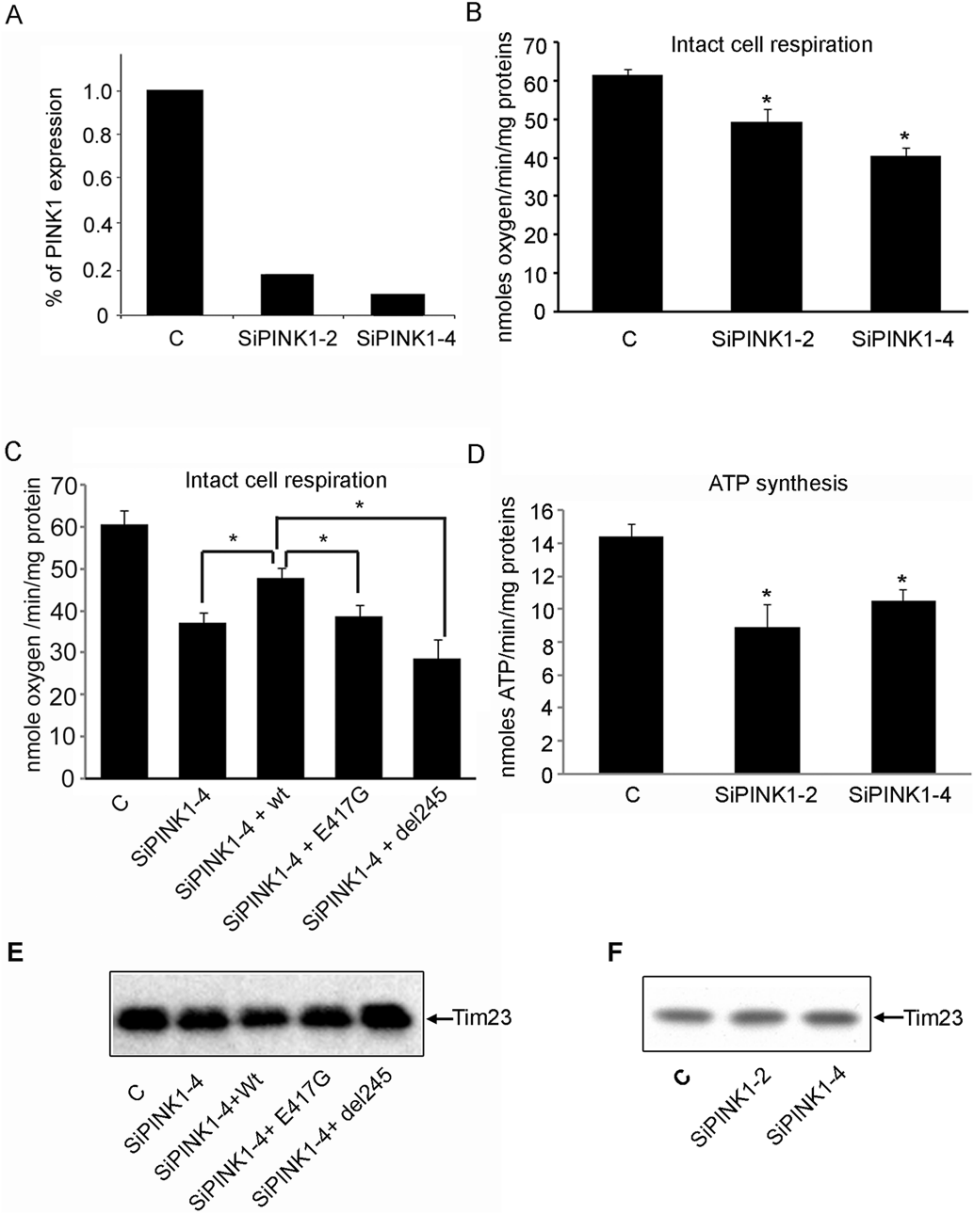
Loss of PINK1 impairs OXPHOS function in PC12 cells with reduced PINK1 expression. Normal respiration is impaired in the PC12 cell lines with PINK1 knocked-down by RNAi. A) PINK1 mRNA is significantly reduced in two stable cell lines expressing PINK1 siRNA. Compared to the wild type control, there is an 81.7% and 91% reduction in PINK1 mRNA in SiPINK1-2 and SiPINK1-4 cell line. PINK1 mRNA is significantly reduced in two stable cell lines expressing PINK1 siRNA. The level of PINK1 mRNA is normalized to GAPDH. B) Oxygen consumption is significantly reduced in both SiPINK1-2 (22.4% reduction, n = 10, p<0.05, ANOVA) and SiPINK1-4 (33.1% reduction, n = 11, p<0.01, ANOVA) cell lines compared to that of control cells. SiPINK1-2: 46.38±3.5SE; SiPINK1-4: 39.96±1.93SE; control cell: 59.70±2.1SE. C) The respiratory deficit in SiPINK1-4 cells can be partially rescued by wild type (n = 7, p = 0.008, student T test) but not E417G-PINK1 (n = 4, p = 0.76, student T test) or Del 245 PINK1 (n = 3, p = 0.1, student T test). Control: 60 ±3.38SE; SiPINK1-4: 37±2.32SE; SiPINK1-4/wt-PINK1: 47.9±2.39SE; SiPINK1/E417G: 38.53±3.35SE; SiPINK1/Del 245: 28.63±4.75SE. D) With glutamate/malate as substrates, ATP synthesis rate was significantly reduced in both SiPINK1-2 (41.3% reduction, n = 7, p<0.01, ANOVA) and SiPINK1-4 (29.8% reduction, n = 8, p<0.01, ANOVA) cell lines compared to that of control cell. SiPINK1-2: 8.73±0.99SE; SiPINK1-4:10.59±0.69SE; control cells: 14.883±0.78SE. E) Western analysis of the samples used for the rescued experiment shown in (C) with Tim 23 Ab. The result demonstrates that equal amount of mitochondria is present in all the samples subject to respiration experiment. F) Equal amount of mitochondria were used for the experiments shown in B and D as demonstrated by identical Tim 23 in all the samples.

In order to elucidate whether the OXPHOS deficit observed above was the result of a PINK1 defect, rather than an artifact of protein over-expression, we knocked-down PINK1 expression by 80% in PC12 cells stably transfected with siRNA against PINK1 (**Figure 6A**). PINK1 knockdown led to a statistically significant reduction in oxygen consumption (**Figure. 6B**), similar to expression of mutant PINK1 (**Figure 5A**). Importantly, this respiration deficit could be partially rescued by wild type PINK1 but not by mutant PINK1, further demonstrating that the respiration deficit is specifically due to mutant PINK1 (**Figure 6C**).

Consistent with the observation described in **Figure 5B**, a deficit in ATP synthesis rate was detected in mitochondria isolated from PINK1 knock-down (KD) cells (**Figure 6D**). Since mitochondrial quantity was normalized between control and PINK1-KD cells as demonstrated by Western analysis with Tim 23 Ab (**Figure 6E and 6F**), we conclude that the defect in respiration and ATP synthesis seen in the cells with reduced PINK1 is a functional deficit and not due to a sheer reduction of mitochondria. This result also suggests that the decline in steady-state ATP levels previously reported in flies [**41**] is likely due to defective mitochondrial ATP synthesis.

Thus, taken together, OXPHOS function can be impaired by either mutant PINK1 or loss of PINK1.

### Proteasome deficits caused by mutant PINK1 or loss of PINK1

Among the many cellular functions that require ATP, proteasomal activity is particularly important for PD. PD pathology is generally characterized by the presence of SDS-insoluble protein inclusions of α-synuclein. These inclusions are often positive for polyubiquitin, implicating a possible impairment of the ubiquitin proteasome system (UPS). The UPS comprises a tightly regulated succession of steps that first tag proteins with a polyubiquitin chain, then target the proteins for degradation by the 26S proteasome [42]. Covalent attachment of the polyubiquitin chain is dependent upon a series of ubiquitin ligases E1, E2 and E3 that systematically bind ubiquitin to its substrate in an ATP-dependent manner. The tagged protein is then rapidly degraded by the 26S proteasome, which requires ATP to assemble from the 19S and 20S subunits. The importance of the UPS in PD is exemplified by the identification of mutations in Parkin, an E3 ligase, as the cause of autosomal recessive PARK2 form of PD.

**Figure 7:**
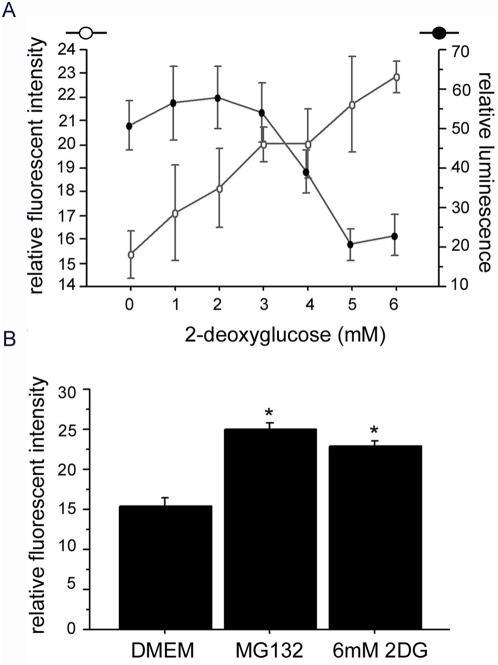
Proteasome function is impaired by reduction of ATP. Proteasome function is ATP dependent. Fluorescent CFP was fused to degron, a signaling peptide that directs its protein to proteasome for degradation. An increase of fluorescence (open circle) indicates a reduction of proteasome function. ATP production was inhibited by 2-deoxyglucose (2DG), and ATP content was measured with the ATP Assay Kit (Calbiochem) for luminescence (filled circle). A) Increasing dosages of 2DG caused a decrease in ATP production (filled circle) and enhanced proteasome inhibition (open circle). B) Compared to non-treated cells in DMEM, there is a significant proteasome inhibition by 6 mM 2DG (p = 0.0004, ANOVA) and the proteasome inhibitor MG132 (p = 0.0001, ANOVA).

Since mutant and PINK1 KD cells evidenced a significant ATP production deficit (**Figure 5B** **and** **6D**), we decided to study whether this ATP deficit which is induced by loss of PINK1 or mutant PINK1 could also influence the proteasome function. To pursue this line of investigation, we first examined whether ATP affects proteasome activity in our experimental systems. We treated SH-SY5Y and HeLa cells with 2-deoxyglucose (2DG) to decrease ATP levels via inhibition of glucose utilization. In these cells, proteasome degradation of a degron fused monomeric CFP (mCFP-degron) [43] was diminished in a 2DG dose-dependent manner (**Figure 7A, 7B**). Therefore, in our systems, progressive ATP depletion decreases proteasome activity, consistent with the observation in other cell culture systems [**44**].

**Figure 8:**
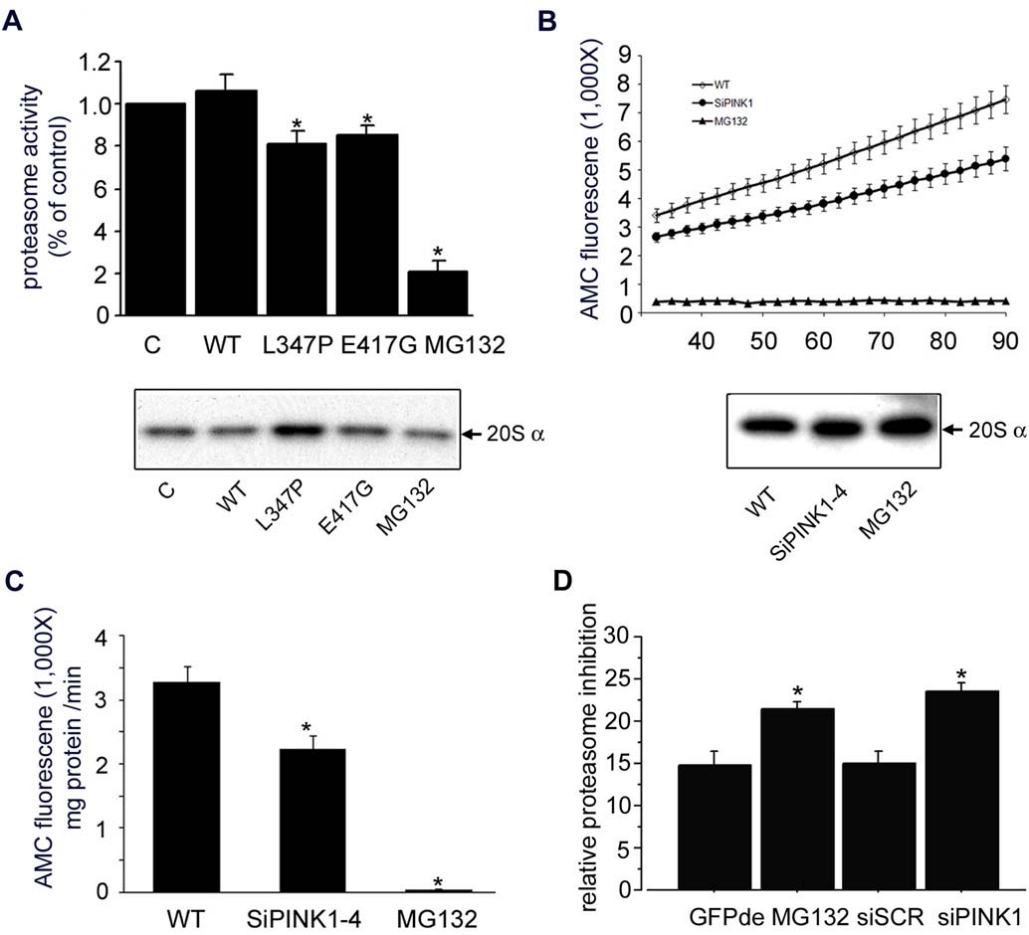
Proteasome function is impaired by mutant or loss of PINK1. Proteasome activity was measured from SH-SY5Y cells expressing mutant PINK1 (A), and PC12 cells expressing siRNA against PINK1 (B, C, D). A) Fluorescence of fluorogenic proteasome substrate Suc-LLVY-AMC (Calbiochem) is positively correlated with proteasome function. No statistically significant changes were detected in proteasome activity between control SH-SY5Y cells and the cells expressing wild type PINK1 (n = 8, p = 0.484, paired student t test). There was a statistically significant decrease of proteasome activity in the SH-SY5Y cells expressing L347P-PINK1 (23% reduction, n = 7, p = 0.018, paired student t test) or in SH-SY5Y cells expressing E417G-PINK1 (19.4% reduction, n = 8, p = 0.012, paired student t test) compared to cells expressing wild type PINK1. MG132, a proteasome inhibitor, was used as a negative control. The bottom panel is a Western analysis of the above samples with the 20S α subunit Ab for normalization. B) Proteasome activity was measured in 20 µg of cell lysate isolated from wild type control PC12 cells (open diamond) or SiPINK1-4 PC12 cell line (filled circle) for 60 min after 30 min incubation. Wild type PC12 cells lysate treated with MG132 (filled triangle) was used as a negative control. The result revealed that the kinetic of proteasome activity monitored over 60 min was markedly decreased in the cells with reduced PINK1. The bottom panel is a Western analysis of the above samples with the 20S α subunit antibody for normalization. C) Histographic presentation for Figure 7B. The reduction of PINK1 by siRNA impairs the proteasome activity (31.8% reduction, n = 8, p = 0.01, ANOVA). Experiments were repeated with SiPINK1-2 PC12 cell line, and consistent results were obtained (data not shown). D) PINK1 mediated proteasome activity deficit confirmed by another independent method in the HeLa cells. Compared to control (CFP-de transfection), siRNA against PINK1 (siPINK1) knocked down PINK1 and led to a sigfinicant inhibition of CFP degradation (p = 0.0001, ANOVA) to an extent similar to direct proteasome inhibition by MG132 (p = 0.0014, ANOVA). A scrambled siRNA (siSCR) had no effect (p = 0.876, ANOVA). The RNAi sequences are: GAGAGGUCCAAGCAACUA TT and CCUGGUCGACUACCCUGAU TT.

Next, cells over-expressing L347P-PINK1 and E417G-PINK1 were assessed for proteasome function. Consistent with their effect on ATP production, L347P-PINK1 and E417G-PINK1 led to decreased degradation of the proteasome substrate Suc-Leu-Leu-Val-Tyr-AMC, which was reflected by decreased fluorescence (**Figure 8A**). The wild type PINK1 overexpression did not lead to a deficit in proteasome function, indicating that the effect of L347P-PINK1 and E417G-PINK1 on proteasome was not due to protein overexpression *per se*. By controlling and normalizing for the proteasomal 20S α subunit, we conclude that the proteasome deficit is a functional deficit rather than a decrease of proteasome (**Figure 8A, bottom panel**).

To further prove that the proteasome dysfunction is the result of a PINK1 defect, we examined the proteasome function in PINK1 depleted cells. PINK1 KD led to a statistically significant reduction in proteasome function (**Figure. 8B and 8C**), similar to expression of mutant PINK1 (**Figure 8A**). Proteasome amounts were comparable between control and PINK1 depleted cells, as demonstrated by Western analysis with proteasomal 20S α subunit Ab (**Figure 8B, bottom panel**), suggesting that the defects observed in proteasome activity upon PINK1 depletion may be due to a functional deficit rather than a decrease in the amount of proteasome. To further prove the deleterious effects of knocking down PINK1 on the proteasome function, we measured proteasome activity by proteasome-mediated degradation of mCFP-degron, corroborating that PINK1 KD significantly impairs proteasome function (**Figure 8D**).

### α-synuclein aggregation

Abnormal protein accumulation and aggregation are common features in neurodegenerative diseases. In PD, α-synuclein-containing Lewy body pathology is present in most cases, and PINK1 protein is found in Lewy bodies [45], [46]. An important factor for α-synuclein accumulation and aggregation is whether the protein degradation pathway is functioning properly. In fact, transgenic mouse models for α-synuclein have defective proteasome function [47].

**Figure 9:**
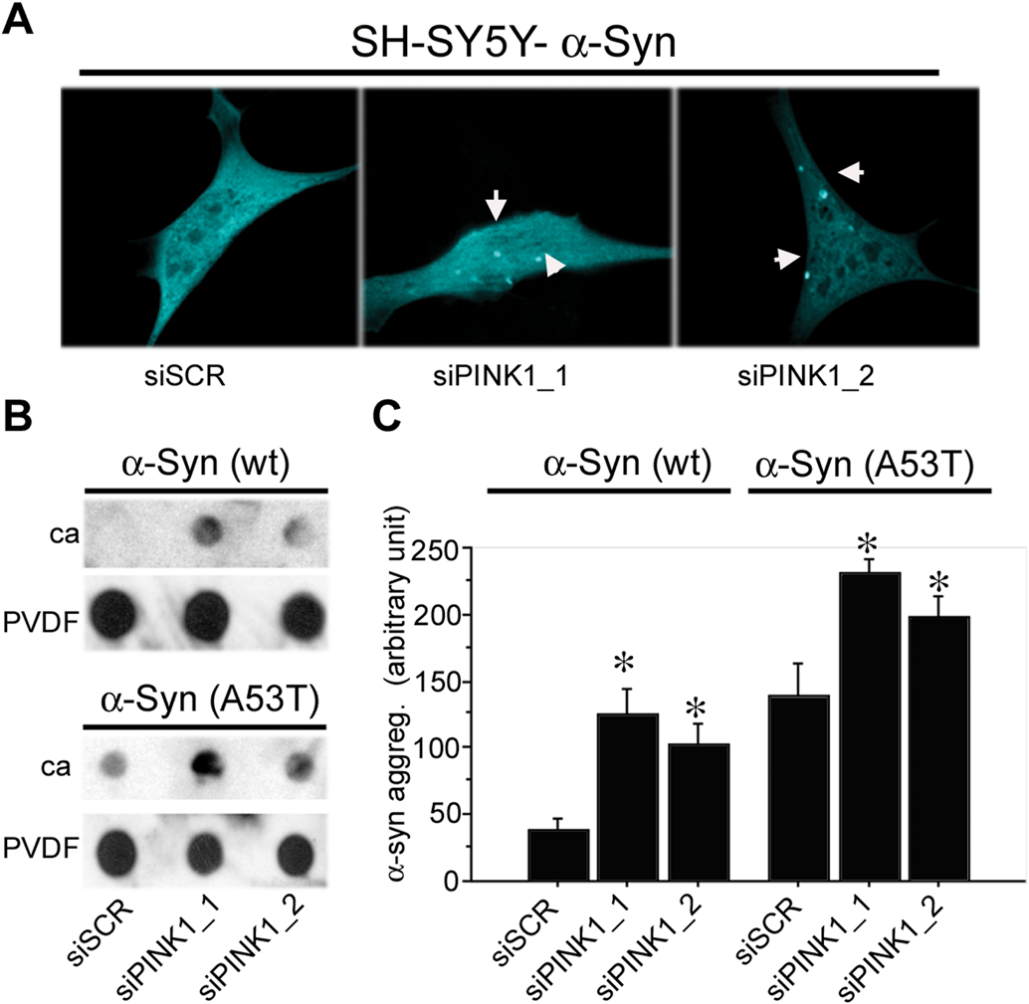
Loss of PINK1 function leads to α-synuclein aggregation. Knockdown of PINK1 by siRNAs in cell lines expressing wild type α-synuclein-mCFP or A53T α-synuclein-mCFP leads to the accumulation and aggregation of α-synuclein as measured by confocal microscopy or filter trap assay. A) Representative confocal images of SH-SY5Y cells co-transfected with α -synuclein-mCFP and siRNA. The results indicate that loss of PINK1 leads to the formation of mCFP-positive inclusions. B) Dot blot filter trap assay captured SDS-insoluble inclusions formed by loss of PINK1 function. Two different siRNA with sequences against PINK1 (siPINK1-1 and siPINK1-2) were transfected into stable HeLa cell lines expressing wt α-synuclein or A53T α-synclein. The lysate was collected and filtered through two filters, with the cellulose acetate (ca) membrane on the top and the PVDF membrane at the bottom. The ca membrane trapped the insoluble aggregates, whereas PVDF membrane caught the soluble protein. The results indicate that reduction of PINK1 by either siRNA leads to increased accumulation of SDS insoluble α-synuclein compared with the scrambled siSCR control when filtered through ca membrane. PVDF membrane was blotted with actin as loading control. C) Quantification of filter trap assay using densitometry analysis via NIH Image. Each bar represents 4 samples. siRNA-mediated knockdown of PINK1 leads to a significant increase of SDS-insoluble α-synuclein (*: p<0.05). ANOVA analysis reveals a significant effect of α-synuclein (F_(1,18)_ = 56.482; p<0.001) and siRNA (F_(2,18)_ = 15.559; p<0.001), but no significant interaction between the two variables (F_(2,18)_ = 55.413; p = 0.949), thus indicating that PINK1 knockdown has a similar effect on both forms of α-synuclein.

Since the loss of PINK1 function impairs proteasome-mediated degradation (**Figure 8**), we next examined if it would promote the formation of SDS-insoluble inclusions of α-synuclein. Co-transfection of α-synuclein-mCFP with either of the two different siRNA against PINK1 (siPINK1) in SH-SY5Y cells led to the formation of mCFP-positive inclusions as revealed by confocal microscopy. A control scramble sequence siRNA (siSCR) led to no visible aggregates (**Figure 9A**). The same results were also obtained in stable cell lines that express α-synuclein-mCFP at low levels (data not shown).

We next used a modified filter trap method to better quantify the amount of SDS-insoluble aggregates (**Figure 9B and 9C**) [48]. Loss of PINK1 led to increased SDS-insoluble aggregates in both the wild type α-synuclein and the human disease form A53T-α-synuclein. Taken together, these results show that loss of PINK1 is sufficient to lead to a decrease of proteasome function and accumulation of insoluble α-synuclein aggregates.

## Discussion

In an effort to dissect the molecular pathway for PINK1 mediated pathogenesis, we have obtained new insights into the nature of mitochondrial dysfunction and its deleterious cellular consequences.

The mitochondrial OXPHOS system is known to be regulated by kinases. We have demonstrated that mutant PINK1 or loss of PINK1 cause deficits in respiration and ATP synthesis. Similar and yet different observations were made in other systems. In *Drosophila*, mitochondrial structural defect accompanied by reduced ATP content were reported [23], [49]; Additionally, complex I and II driven respiration deficits were also detected in mice, though surprisingly, this was in the absence of any ATP deficit [21]. While the molecular details need to be elucidated, it is likely that PINK1 mediated defects are caused by changes in the phosphorylation states of ETC Complexes. In support of this notion, it is known that several subunits of mitochondrial ETC Complexes are phospho-proteins, and their activity can be regulated via PINK1 mechanism [50], [51].

It is important to note that PINK1-induced deficit in ETC and subsequent cellular dysfunctions are similar to those induced by rotenone toxicity in rats [10]. Pharmacological inhibition of the ETC, particularly Complex I, result in PD-like phenotypes, but how these models reflect actual PD pathogenesis remains to be elucidated. Our results offer a possible link between specific mitochondrial dysfunctions and cellular abnormalities that are highly relevant to PD.

We found that proteasome function is impaired by mutant or reduced amounts of PINK1. The proteasome is one of the major pathways for protein degradation. Parkin, the disease gene for PARK2 type of PD, encodes an E3 ubiquitin ligase. One of Parkin's proposed roles is the proteasomal degradation of its protein substrates. In *Drosophila*, it was shown that Parkin and PINK1 have a genetic interaction [49], [52]. It will be interesting to investigate if the interaction between Parkin and PINK1 occurs in mitochondria, and whether it affects mitochondrial function.

Our results identified concomitant deficits in mitochondrial bioenergetics, proteasomal activity, and α-synuclein aggregation. Are they consequential to each other or independent events? We postulate that since PINK1 is predominantly localized in mitochondria, the primary pathogenic event is likely to be in the same place. The ATP deficit is a potential link between mitochondrial abnormality and proteasome deficit, although proteasome deficit could also be caused by other mechanisms such as abnormal post-translational modification including phosphorylation, assembly and targeting, etc. α-synuclein is known to be a target of proteasome degradation in the cytosol [53], [54]. Therefore, α-synuclein aggregation could be the consequence of proteasome dysfunction. In addition, since α-synuclein aggregation has been shown to affect proteasome function directly [47], it is tempting to speculate a vicious cycle of proteasomal dysfunction and α-synuclein aggregation, although further experimental data are needed to support this hypothesis.

A common theme in neurodegeneration is that for any given disease-causing mutant protein, a large number of interwoven cellular dysfunctions have been discovered. Our observations start to unravel a subset of PINK1 pathogenic processes, and will certainly lead to other highly relevant pathways. For example, deficits in respiratory complexes identified in our experiments, in addition to bioenergetic impairment, may also lead to increased oxidative stress, as was shown in rotenone models and other studies [10], [55]. Furthermore, the ATP deficit is likely to have a much wider negative impact on many cellular functions in addition to proteasome activity. Clearly, it will be important to further assess as many mitochondrial and other cellular functions as possible to dissect the full spectrum of PINK1 pathogenesis. As this manuscript was reviewed, Gautier et al reported respiration deficit in the PINK1 knockout mice [21]. Our data, not only show similar decrease in respiration, but also pointed out the downstream deleterious consequence of this deficit, such as decrease in ATP synthesis rate, proteasomal deficit, and α-synuclein accumulation. Therefore, our results provide a framework for PINK1 mediated pathogenesis, upon which future studies can be designed and pursued.
